# SARS-CoV-2 Cluster in Nursery, Poland

**DOI:** 10.3201/eid2701.203849

**Published:** 2021-01

**Authors:** Magdalena Okarska-Napierała, Joanna Mańdziuk, Ernest Kuchar

**Affiliations:** Medical University of Warsaw, Warsaw, Poland

**Keywords:** COVID-19, coronavirus disease, SARS-CoV-2, severe acute respiratory syndrome coronavirus 2, viruses, respiratory infections, zoonoses, toddlers, infectiousness, daycare, transmission

## Abstract

We report a cluster of surprisingly high spread of severe acute respiratory syndrome coronavirus 2 (SARS-CoV-2) associated with a single nursery in Poland. Our findings contrast with the presumed negligible role of children in driving the SARS-CoV-2 pandemic. Children 1–2 years of age might be effective SARS-CoV-2 spreaders.

Despite robust research, knowledge about coronavirus disease (COVID-19) spread and effective control measures is still limited. Until recently, research has indicated that children rarely spread the infection to adults and are not the primary drivers of severe acute respiratory syndrome coronavirus 2 (SARS-CoV-2) transmission (*1*).

We describe characteristics of the cluster of SARS-CoV-2 cases that emerged in a single nursery in Poland within 2 weeks of its reopening. We anonymized all data and collected no sensitive data. The Bioethics Committee of the Medical University of Warsaw approved the study protocol.

The nursery at issue was reopened after a nationwide lockdown on May 18, 2020. On May 31, a nursery worker reported family contact with a symptomatic SARS-CoV-2–infected person, and the nursery was closed. During the 14 days the nursery was open, a mean of 25 children attended the nursery daily. Children spent »8 hours there, divided into 3 groups, each cared for by 2 caregivers (Appendix). Neither children nor caregivers moved across multiple classes. Caregivers wore facemasks when in contact with children. Parents did not enter the building when dropping off and picking up children. Contacts between parents and nursery workers lasted <15 minutes, with facemasks on. Family members of different children did not mix.

The index case of SARS-CoV-2 infection (in a nursery worker with family contact) was confirmed on June 4. Subsequent PCR testing of nursery staff, children attending the facility, and family members (2 initial case-patients plus 104 other persons) (Appendix) revealed positive results in an additional 4 nursery workers (of whom 1 was also a parent of a child attending the facility), 3 children of the nursery workers, 8 children attending the facility, 3 siblings of those children, 8 parents, and 1 grandparent. The cluster involved a total of 29 persons; 8 were children attending the nursery, and 12 were children’s family members who did not enter the facility (Table). One child with a negative result had 2 parents with positive results. One child’s parent tested negative in this cluster but had tested positive within the previous 2 weeks, involved in another cluster.

**Table:** Severe acute respiratory syndrome coronavirus 2 testing outcomes of potentially infected persons in a nursery setting, Poland, 2020

Person	No. tested	No. positive by PCR	% Positive
Children attending the nursery	28	8	29
Parents of children attending nursery*	31	8	26
Siblings of children attending nursery	16	3	19
Grandparent of children attending nursery	1	1	100
Nursery workers	25	5	20
Spouses of nursery workers	2	1	50
Children of nursery workers	3	3	100
Total	106	29	27

*One of the parents of children attending the nursery was also a nursery worker; she is counted as a worker in this table.

The overall positivity rate in our cluster was 27%. COVID-19 prevalence in Poland is low. The number of tests conducted in the country was 124,194 in June, whereas the number of positive cases was 1,374, which corresponded to a positivity rate of 1% (*2*). Thus, local SARS-CoV-2 circulation in society is not sufficient to explain the positivity rate in our cluster. The case of the COVID-19–negative child with positive parents could have been a false-negative result or a negative result after being infected. The result might also have been a true negative, and the parents were infected from another source. However, other potential exposures could not explain infections in all parents involved in our cluster.

We depict probable chains of transmission in the Figure. Of note, physical contact between nursery workers and children’s family members who were infected was strictly limited, and the only close contacts for these groups of adults were children. Given that most COVID-19–positive persons were asymptomatic and tested on the same day, determining with certainty whether children transmitted the virus to their parents or the workers is not possible. Nevertheless, children seemed to be effective mediators of infection between adults.

**Figure:**
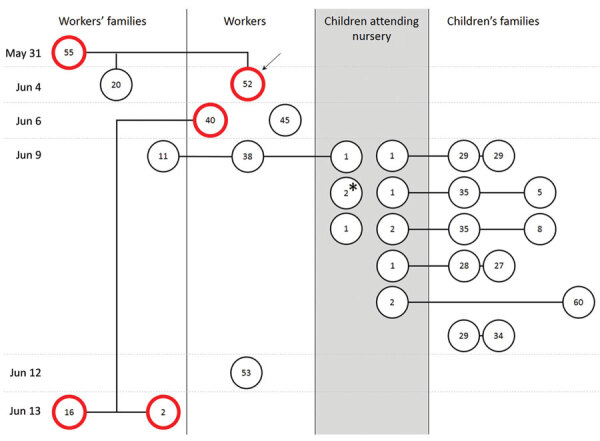
All persons testing positive for severe acute respiratory syndrome coronavirus 2 infection in a cluster associated with a nursery, Poland 2020. Dates to left indicate first positive results in consecutive case-patients. Circles indicate infected case-patients; numbers in circles indicate age in years. Red circles indicate infected case-patients with symptoms. Circles connected by lines indicate case-patients who are members of the same household. Arrow indicates the probable index case-patient. Asterisk indicates child whose parent tested negative in this cluster but tested positive within the previous 2 weeks.

Several reports concerning clusters of COVID-19 in childcare settings imply little to no SARS-CoV-2 transmission among children and from children to adults (*1*,*3**–**5*; A. Fontanet, unpub. data, https://doi.org/10.1101/2020.06.25.20140178; R.M. Viner, unpub. data, https://doi.org/10.1101/2020.05.20.20108126). However, such estimations are open to bias, given that most published data were obtained at the time of lockdown, when children’s social contacts were limited to family members. Another limitation of those publications is that they applied mostly to school-age children.

The high infection attack rate among children in our cluster could be explained by prolonged close contact between very young children, who are less able to adjust to control measures. Similarly, specific intimate contact between toddlers and their family members could have led to effective spread within families. This observation might be particularly important in light of novel findings that nasopharyngeal SARS-CoV-2 levels are the highest in the youngest children (*6*). Moreover, the airborne transmission route in the nursery rooms’ confined environment could have played an important role (*7*).

Our study has some potential limitations. We could not determine whether the infection in the nursery worker was the real index case because one of the children’s parents had tested positive within the previous 2 weeks and that child could also have been the primary case. Moreover, we could not verify the information we obtained from the nursery about the facility’s prevention methods.

Our report questions the role of young children in driving the COVID-19 pandemic. Of note, most children in our study were asymptomatic, and this cluster would likely not have been detected without subsequent testing of persons who had direct contact with the index case-patient. We believe further studies are needed to clarify young children’s role in the transmission of SARS-CoV-2.

Appendix Additional information about SARS-CoV-2 cluster in nursery, Poland.

## References

[1] Ludvigsson JF. Children are unlikely to be the main drivers of the COVID-19 pandemic - A systematic review. Acta Paediatr. 2020;109:1525–30. 10.1111/apa.15371 32430964 PMC7280674

[2] Rogalski M. COVID-19 w Polsce. Database based on Polish Ministry of Health reports [cited 2020 Aug 26]. http://bit.ly/covid19-polska

[3] Li X, Xu W, Dozier M, He Y, Kirolos A, Theodoratou E; UNCOVER. The role of children in transmission of SARS-CoV-2: A rapid review. J Glob Health. 2020;10:011101. 10.7189/jogh.10.011101 32612817 PMC7323934

[4] Yung CF, Kam KQ, Nadua KD, Chong CY, Tan NWH, Li J, et al. Novel coronavirus 2019 transmission risk in educational settings. Clin Infect Dis. 2020;ciaa794. 10.1093/cid/ciaa794 PMC7337629 32584975

[5] Somekh E, Gleyzer A, Heller E, Lopian M, Kashani-Ligumski L, Czeiger S, et al. The role of children in the dynamics of intra family coronavirus 2019 spread in densely populated area. Pediatr Infect Dis J. 2020;39:e202–4. 10.1097/INF.0000000000002783 32496407

[6] Heald-Sargent T, Muller WJ, Zheng X, Rippe J, Patel AB, Kociolek LK. Age-related differences in nasopharyngeal severe acute respiratory syndrome coronavirus 2 (SARS-CoV-2) levels in patients with mild to moderate coronavirus disease 2019 (COVID-19). JAMA Pediatr. 2020;174:902–3. 10.1001/jamapediatrics.2020.3651 32745201 PMC7393583

[7] Morawska L, Cao J. Airborne transmission of SARS-CoV-2: The world should face the reality. Environ Int. 2020;139:105730. 10.1016/j.envint.2020.105730 32294574 PMC7151430

