# Supplementary material for: SARS-CoV-2 Cluster in Nursery, Poland
Source: Emerg Infect Dis. 2021 Jan;27(1):317–9. doi: 10.3201/eid2701.203849 (PMC7774538; doi:10.3201/eid2701.203849)
Supplement: Appendix — Additional information about SARS-CoV-2 cluster in nursery, Poland. [file 20-3849-Techapp-s1.pdf]

Article DOI: https://doi.org/10.3201/eid2701.203849

SARS-CoV-2 Cluster in Nursery, Poland

Appendix

Detailed Characteristics of Children's Groups and Testing Sequence

Children in the nursery were divided into 3 groups according to age (group A, the youngest; group B, medium; group C, the oldest). The mean number of children attending the nursery every day was 10–12 for group A, and 5–7 for groups B and C. The total number of children attending group A was 16, and 12 in both groups B and C.

When the first COVID-19 cases were identified among the nursery workers, children were split into "direct contact" and "indirect contact" groups. Children from group A were considered "direct contacts" and thus were tested together with all their household members. Children from groups B and C were "indirect contacts," and only children were tested. Of note, 1 nursery worker was a parent of a child from group A and worked with children from group B. She tested positive, but all children with whom she worked tested negative, and thus their families were not tested.

The sequence of testing was as follows.

1. The index case, who was a caregiver in group A, tested positive on June 4, together with her adult child.
2. Two more nursery workers working in close contact with the index case tested positive on June 6 (one of them was another caregiver in group A).
3. On June 9, all children from group A (16 children) and all their household members (32 parents, 16 siblings, and 1 grandparent) were tested.
4. The rest of children attending the nursery (groups B and C, 12 children) were tested on consecutive days, and none of them tested positive. Their family members were not tested. Similarly, the rest of the nursery workers, who did not have direct contact with the first 3 cases, were tested on consecutive days.

5. Two more children of one nursery worker were tested 7 days after she tested positive, and they were positive.

Testing practices implemented in the nursery by a local sanitary-epidemiological station complied with standard testing procedures in the country at that time. They involved testing symptomatic people and contacts of confirmed COVID-19 cases.

PCR Testing

The testing was done in a single validated laboratory at the local sanitary-epidemiological station.

The isolation phase of the reaction was performed on Veri-Q Prep M16 Nucleic Acid Extraction Device using Viral DNA/RNA Prep/kit-Airway Sample (Humanitas Global Logistics, https://hglmedical.com/). The amplification phase was performed on Veri-Q PCR 316 Gene Amplification Device using nCoV19 Detection PCR Kit.

Clinical Picture

There were no symptomatic cases among children attending the facility and their family members. A total of 5 people had COVID-19 symptoms. The family member of the index case was diagnosed and treated in the hospital. Two nursery workers and 2 children of 1 of them had mild symptoms of the disease.

Seven children attending the nursery and 2 of their siblings were evaluated in the hospital. Laboratory testing and chest x-ray revealed no abnormalities except for an 11-year-old girl who had elevated transaminase levels (alanine transaminase 297 U/I, aspartate transaminase 167 U/I) that normalized within 1 week.
